# ‘Development and psychometric evaluation of the safety feeling scale in adult patients at hospital: Exploratory sequential mixed method’

**DOI:** 10.1002/nop2.1850

**Published:** 2023-05-28

**Authors:** Sahar Dabaghi, Mitra Zandi, Abbas Ebadi, Abbas Abbaszadeh, Camelia Rohani

**Affiliations:** ^1^ Department of community health nursing, School of Nursing and Midwifery Shahid Beheshti University of Medical Sciences Tehran Iran; ^2^ School of Nursing and Midwifery Shahid Beheshti University of Medical Sciences Tehran Iran; ^3^ Behavioral Sciences Research Center Life Style Institute, Baqiyatallah University of Medical Sciences Tehran Iran; ^4^ Research Center for Life & Health Sciences & Biotechnology of the Police Direction of Health, Rescue & Treatment, Police Headquarter Tehran Iran; ^5^ Nursing and Midwifery School Shahid Beheshti University of Medical Sciences Tehran Iran; ^6^ Department of Health Care Sciences, Palliative Research Center Marie Cederschiöld Högskola, Campus Ersta Stockholm Sweden; ^7^ Community Health Nursing Department, School of Nursing and Midwifery Shahid Beheshti University of Medical Sciences Tehran Iran

**Keywords:** feeling, hospitalization, patient, patient safety

## Abstract

**Aim and Objectives:**

This study aimed to develop and examine psychometric properties of the safety feeling scale (SFS) in adult patients to assess their sense of safety during a hospital stay.

**Design:**

Mixed methods design. A SQUIRE checklist was used.

**Methods:**

This is a study with two phases of scale development and evaluation of the psychometric properties of the scale. In the first phase, the concept of ‘safety feeling’ was analysed using a hybrid model. Thus, a systematic review and then a qualitative study with hospitalized patients (*n* = 31) were conducted by conventional content analysis. In the psychometric phase, factorial validity, reliability, feasibility, and responsiveness of the scale were evaluated by different tests in various samples.

**Results:**

After integrating the results of the systematic review and qualitative study, a scale item pool with 84 items was developed. In the psychometric phase, 12 items with four factors were specified; ‘effective care,’ ‘confidence in the healthcare team,’ ‘emotional enrichment’ and ‘hygienic facilities,’ explaining 51% of the total variance of the scale. They were confirmed by confirmatory factor analysis. Internal consistency and stability of the scale were satisfactory. Feasibility and responsiveness were also acceptable.

## 1 BACKGROUND

Patients are at the core of care in clinical settings. Delivering safe healthcare services to patients is a highly important issue and fundamental principle in healthcare settings (Zabin et al., 2022). According to the World Health Organization, ‘patient safety’ means prevention and reduction of risks, errors and harms that can occur during the health care of the patient. There is no specific theory in nursing about patient safety. But, there are several theories in other disciplines, including ‘Bowlby's Ethical Approach’ in 1973 (Bowlby, 1973), Fry's ‘Safety the Therapeutic Milieu’ in 1987 and Rachman's ‘Safety Signals’ in 1994 (Woody & Rachman, 1994; Fry, 1987). Measurement of patient safety is a key element in securing and delivering high‐quality healthcare services. Also, this is a multi‐step, systematic and multidisciplinary goal in healthcare settings (Dabaghi et al., 2020; Labrague et al., 2022). Providing patient safety facilitates the provision of safe care for both patients and healthcare personnel (Torkaman et al., 2022).

A review of the literature shows that there are various studies on patient safety in hospital settings to prevent or decrease accidents and medical errors. Evidence shows that ‘culture’ in different communities can influence patients’ perceptions and thus patients’ safety, so there is a priority for research in this area (Dabaghi et al., 2020; Ricci‐Cabello et al., 2016). Unfortunately, adverse events and negative consequences for patients in medical settings still occur frequently. It seems that the complexity of the healthcare environment and multiple environmental factors, impact the provision of safe care (Murphy et al., 2021; Shojania & Marang‐van de Mheen, 2020). There are studies on patient safety culture among healthcare providers and influencing factors, such as the relationship between nurse and patient, hand hygiene and falling out of bed (Granel‐Giménez et al., 2022; Wang & Tao, 2017; Waterson et al., 2019). However, patients’ perceptions play an important role in their awareness of the overt and covert problems in healthcare settings (Asiamah et al., 2022; Butler et al., 2022). ‘Being safe’ is not the same as ‘feeling safe (Dabaghi et al., 2020). Therefore, studies were conducted to understand the patients’ experiences and perspectives on the concept of safety (Geraedts et al., 2020; Hernan et al., 2021). Initially, it was assumed that ‘safety’ gains its meaning from the intensive care units (ICUs), which have sophisticated monitoring equipment and patients have severe injuries (Hupcey, 2000; Russell, 1999). Over time, more studies were conducted in various hospital wards, such as surgery and obstetric wards (Dabaghi et al., 2020; Lee et al., 2010; Nilsson, 2014; Vaismoradi et al., 2011). In these studies, the authors tried to define the concept of safety using different qualitative methods. But, there are many disparities in the definitions at interdisciplinary and intradisciplinary level. For instance, many studies have used the terms ‘safety’ and ‘security’ interchangeably, but there is a border between the meanings of these two terms. Clarification of patient safety is an essential prerequisite to produce a valid and reliable culturally appropriate instrument, and then to develop health interventions and to promote patients' safety at clinical settings. However, there are a few instruments for the measurement of the sense of patients’ 'safety in hospitals.

We found only five instruments in this area; the Scale of Security Feeling in medical‐surgical wards (Nadighara et al., 2016), Patient Measure of Safety (POMS) (Giles et al., 2013), Sense of Security in Care—Patients’ Evaluation (SEC‐P) (Igarashi et al., 2012) and the Feelings of Support and Security in cancer care department (Krevers & Milberg, 2014). Appraisal of selected instruments by COSMIN checklist (COnsensus‐based Standards for the selection of health status Measurement Instruments) showed that these instruments are used in specific wards, such as oncology or internal medicine and surgery, and also in different societies. None of them was valid for our context and no study examined all aspects of feeling safe in detail. Moreover, we identified an overlap between the words of safety and security in these studies. Furthermore, most of them used only a review of the literature for development of an instrument. Finally, we concluded that in none of the studies, the psychometric phases of the instrument were fully performed.

Therefore, we decided to conduct a comprehensive study with a multi‐method design. The aim of this study was to develop a valid and reliable general scale to measure safety feelings in hospitalized adult patients in the cultural context of Iran.

## 2 METHODS

This is a mixed‐method study with exploratory sequential design. It was performed in two different phases: scale development and psychometric properties evaluation (Figure S1). This study was accepted by the research ethics committee of the university (Ethics Code XX). All participants were informed about the aim of the study, voluntary participation and the confidentiality of the information. They were also informed that they could withdraw at any time of the study and written informed consent forms were obtained from all of them. The SQUIRE guidelines were used to guide this study (See File S1).

### 2.1 Phase 1: Scale development

In the first phase of the study, a concept analysis was done on the concept of ‘safety feeling’ using a hybrid model by Shwartz‐Barcott and Kim in three steps, consisting of theoretical stage, fieldwork and final analysis (Schwartz‐Barcott, 2000).

#### 2.1.1 Step 1: Theoretical stage

The aim of this step was to explore the features of ‘safety feeling.’ We wanted to answer two questions based on the literature: ‘What is the explicit meaning of safety feeling’? and ‘what is the boundary between “safety feeling” and “security feeling” at hospital?’ In this step, a systematic review was conducted on the qualitative studies which focused on the patients’ perceptions and/or experiences about the features of safety feeling at the hospital. Studies were selected without language restriction if they published until 5 December 2019 in databases of PubMed, Web of Science, Scopus, ProQuest, Embase and Cochrane Library a number of key terms were selected and checked in the title and abstract of the studies, including “Patients’ perception,” “Patients' experiences,” “Patients 'view,” “Patients’ perspective,” “Patients’ expectations,” “Understanding,” “Safety,” “Security,” “Sense of security,” “Sense of safety,” “Feeling safe,” “Feeling secure,” “Feeling of safety,” “Feeling of security,” “Qualitative research,” “Research qualitative,” “Qualitative studies,” “Qualitative study”. Before data analysis, quality of the studies was estimated by the quality assessment tool JBI‐QARI (Institute, 2014). Eligible studies were analysed using Walker and Avant (2005) approach (Walker & Avant, 2005).

#### 2.1.2 Step 2: Field work

The aim of this step was to explore a comprehensive explanation for ‘safety feeling at hospital.’ We wanted to answer two questions: ‘What is the definition of safety feeling at hospital?’ and ‘what are the dimensions of the concept of safety feeling at hospital?’ In this step, a qualitative study was conducted with 31 patients at four university hospitals. All interviews were analysed by Graneheim and Lundman's conventional content analysis method (2004) (Graneheim & Lundman, 2004).

#### 2.1.3 Step 3: Final analysis

The aim of this step was to integrate the data which obtained from the previous steps (systematic review and qualitative study). The results of the systematic review and qualitative study were integrated, and then a scale item pool was produced.

### 2.2 Phase 2: Psychometric properties evaluation

In the second phase, the validity and reliability of the scale were examined by different tests and samples.

#### 2.2.1 Face validity

Face validity of the scale was assessed through quantitative and qualitative methods. Quantitative face validity was computed by determining the item impact scale (IIS) for each item of the scale with a satisfactory level of ≥1.5 (Polit & Yang, 2016; Waltz et al., 2010). Ten patients were asked to evaluate and score the importance of each item on a 5‐point Likert scale. Next, qualitative face validity of the scale was evaluated by 10 patients. Patients read items and released their comments on the simplicity and clarity of them.

#### 2.2.2 Content validity

Content validity of the scale was evaluated in both qualitative and quantitative methods. In the qualitative method, the scale was assessed based on the grammar, wording, item allocation, and scaling indices by 10 experts’ opinions. The quantitative content validity was evaluated by content validity ratio (CVR), item‐level content validity index (I‐CVI), scale‐level content validity and the index/averaging calculation method (S‐CVI). A panel of specialist experts (*n* = 15) was asked to determine the CVR by assessing the importance of each item using a three‐point rating scale. The panel included methodological study experts and specialists working in the field of patient safety. Then, the CVR of each item was computed according to Lawshe's Table with the minimum acceptable value of 0.49 (Lawshe, 1975; Polit & Yang, 2016; Wilson et al., 2012). I‐CVI was measured by 10 experts who assessed the relevance of each item based on a four‐point Likert scale. For measuring I‐CVI, the number of experts who gave a rating of three or four to the items by the total number of experts was divided. The acceptable CVI for total scale (S‐CVI/Ave) was considered 0.80. But, for items, if there was an item with I‐CVI less than 0.78, it was removed (Polit & Yang, 2016).

#### 2.2.3 Item analysis

After evaluation of the content validity of the scale, item analysis was performed to examine the internal consistency of the scale and to find inappropriate items. The scale was given to 30 patients similar to the main study sample (inclusion criteria: Hospitalized conscious patients aged over 18 years in in‐patient wards of selected university hospitals for at least 3 days, able to speak in Persian and verbalize their perceptions). The initial reliability of the scale was first calculated using Cronbach's alpha coefficient. Then, the correlation of each item with other items was examined by the loop method. It should be a correlation between one item and at least two other items in the scale. A correlation of 0.30 or higher is accepted, otherwise, the item should be removed or revised. Moreover, items with a high correlation (*r* ≥ 0.70), should be merged into one item, if possible (DeVellis, 2016; Polit & Yang, 2016).

#### 2.2.4 Construct validity

The factorial validity of the scale was assessed by two methods as follows: exploratory factor analysis (EFA) and confirmatory factor analysis (CFA). For EFA, the scale was delivered to a convenience sample of the patients (*n* = 300) who met the inclusion criteria of the study (see above, item analysis). The appropriateness of the sample size was examined using the Kaiser–Meyer–Olkin (KMO) test (≥0.80<) as well as Bartlett's test of sphericity. Items with the amount of communalities of 0.40 and above are suitable to enter to the factor analysis (Kellar & Kelvin, 2013; Keszei et al., 2010; Munro, 2005). The eigenvalues of one and higher and the scree plot were used to determine the number of extracted factors. To determine the structure of the scale, the EFA with the method of Maximum Likelihood and Promax rotation was assessed (First, we used varimax rotation with the assumption of independence of factors. Because the cross‐loading was high and the arrangement of the items in the factors could not be explained, we used other methods, which became more logical and explainable with the Promax rotation). (Kellar & Kelvin, 2013; Keszei et al., 2010; Munro, 2005).

In the next step for evaluation of the CFA, the scale was distributed among a new convenience sample of the patients (*n* = 200) who met the inclusion criteria of the study (see above, item analysis). CFA was performed using structural equation modelling (SEM). Nine fit indices were considered for estimated parameters, including CMIN/DF (minimum discrepancy function by degrees of freedom divided), RMSEA (root mean square error of approximation), RMR (root mean square residual), PNFI (parsimonious normed fit index), NFI (normed fit index), CFI (comparative of fit index), GFI (goodness of fit index), IFI (Tucker–Lewis index) and AGFI (adjusted goodness of fit index). In both EFA and CFA, items with factor loadings of ≥0.30 remained in the scale (Polit & Yang, 2016).

#### 2.2.5 Reliability

The reliability of the scale was measured using three approaches in three different convenient patient groups (*n* = 30). These approaches were as follows: internal consistency (criteria: *r* ≥ 0.70), intraclass correlation coefficient (ICC) (criteria: ICC ≥ 0.80) and standard error of measurement (criteria: the lower the value, the greater the reliability) (Hu & Bentler, 1998; Polit & Yang, 2016; Schreiber et al., 2006).

#### 2.2.6 Feasibility

The feasibility of the scale was evaluated using floor‐ceiling effect and the average time to complete the scale (DeVellis, 2016; Grove et al., 2012; LoBiondo‐Wood et al., 2018).

#### 2.2.7 Responsiveness

For measurement of responsiveness, minimum detectable change (MDC) in the scale items was measured. MDC below 10% was considered excellent and less than 30% was considered satisfactory. MDC was measured by the following formulas (Polit & Yang, 2016).

$${\mathrm{MDC}}_{95}=\mathrm{SEM}\times \sqrt{2\;}\times 1.96$$

$$\mathrm{MDC}\%=\left(\mathrm{MDC}÷\text{mean}\right)\times 100$$

### 2.3 Data analysis

The data were analysed using SPSS version 20 (IBM Corporation). Also, Lisrel version 8.8 was used for CFA analysis. Both item‐ and subscale‐level analyses were conducted using descriptive statistics including frequencies, means and standard deviations. The normality of the data, skewness, kurtosis and outliers were assessed. The data showed to have a normal distribution. The data were treated and outliers were removed and missing values were replaced by the series mean in the SPSS version 20. In both EFA and CFA, the amount of missing values was acceptable (less than 5%).

## 3 RESULTS

### 3.1 Scale development results in phase 1

#### 3.1.1 Theoretical stage results

After the systematic review in response to the explicit meaning of the safety feeling, 11 main categories were extracted from 25 reviewed qualitative studies: ‘receiving safe care,’ ‘appropriate physical environment,’ ‘resorting to spirituality,’ ‘having previous negative experiences,’ ‘presence of family and friends,’ ‘feeling of protection in a safe place,’ ‘emotional enrichment and confidence,’ ‘comfort and tranquility,’ ‘feeling of control on the situation,’ ‘optimism towards life’ and ‘coping.’ Also, the results showed that the concept of ‘safety’ is beyond ‘security,’ and it is necessary to have security in order to feel safe.

#### 3.1.2 Fieldwork results

In response to the definition of safety feeling at hospital and its dimensions, the results of our qualitative content analysis showed three main categories: ‘feelings of fear and apprehension,’ ‘insolvency’ and ‘trying to retrieval welfare.’ The feeling of safety at hospital is a relative concept with three dimensions and it is perceived by the feeling of protection in a safe place, emotional richness and confidence in the healthcare team and lack of anxiety. It is defined by the needs and expectations met, having positive experiences, receiving human care, family support, a relaxing environment and recourse to spirituality has been created and leads to a feeling of peace, having a sense of control over the situation, hope for life, adaptability and comfortable spending of hospitalization days. The detail of the results has been published elsewhere (Dabaghi et al., 2020).

#### 3.1.3 Final analysis and scale items pool results

After integrating the results of the systematic review and qualitative study, four main categories were created in the last step of the content analysis. Four main categories for the concept of safety feeling at hospital were as follows: ‘feeling of protection in a safe place,’ ‘lack of concern,’ ‘emotional enrichment’ and ‘confidence in the healthcare team.’ Afterwards, items were extracted from the main categories and a scale items pool with 84 items was produced in the research team.

### 3.2 Psychometric properties evaluation results in phase 2

#### 3.2.1 Face and content validity results

In the qualitative face validity of the scale, two items with low impact scores were removed (questions number 1; IIS = 0.68 and question number 2; IIS = 0.57). After the qualitative face validity, six items of the scale were revised according to the patients’ idea.

For qualitative content analysis, the scale was assessed by 10 experts’ opinions and a mild revision was done on the grammar and wording of several items. Based on the opinions of a panel of 15 experts for estimation of the scale CVR, 38 of 84 items were removed and leaving 46 items. Removed items showed CVR between 0 and 0.33 (less than 0.49 in Lawshe's Table).

Subsequently, based on the minimum acceptable value of CVI for each item, two items with values of 0.2 and 0.5, were removed, and thus 44 items remained in the scale. Also, S‐CVI/Ave of the scale was measured to be 0.90.

#### 3.2.2 Item analysis results

At this stage, items 21 (separate hospitalization of male and female patients) and 33 (honesty of the medical staff with the patient) were removed because the level of the standardized Cronbach's alpha coefficient of the scale items increased after deletion of these items and reached to 0.925. Two items with correlation of more than 0.70 combined to one item (items number 7 and 8). Two items of 13 and 42 were revised from negative to positive wording due to the patients' misconceptions. Also, two items (items number 18 and 22) were removed due to the excessive overlap with other items, then 39 items remained in the scale. In the next step, the level of the standardized alpha coefficients for items was calculated again. It showed that it was not increased after item deletion. However, 12 items still showed an item‐total correlation of less than 0.30 (range: −0.52 to 0.25), thus they were removed and the rest of the items were re‐checked by the research team and at last 27 items remained in the scale.

#### 3.2.3 Construct validity results

The demographic characteristics of the samples for both the EFA and CFA are presented in Table 1. The mean age of the patients (Mean ± SD) for EFA and CFA was 48 ± 18.3 and 48.9 ± 17.6 years old respectively.

**TABLE 1:** Demographic characteristics of the sample in the EFA and CFA (*n* = 500).

Variables	EFA (*n* = 300) *n* (%)	CFA (*n* = 200) *n* (%)
Age (year)		
≤30	49 (16.3)	22 (11)
31–50	125 (41.7)	95 (47.5)
>51	126 (42.0)	83 (41.5)
Sex		
Female	163 (54.3)	110 (55)
Male	137 (45.7)	90 (45)
Marital status		
Married	238 (79.3)	154 (77)
Single	48 (16.0)	32 (16)
Divorced/Widowed	14 (4.7)	14 (7)
Educational level		
Elementary school or less	93 (30.0)	69 (34.5)
Secondary & high school	59 (19.7)	26 (13)
Diploma	85 (28.3)	49 (24.5)
University	63 (21.0)	56 (28)
Job		
Employed & disabled	97 (32.3)	82 (41)
Unemployed	36 (12.0)	23 (11.5)
Housewife	122 (40.7)	67 (33.5)
Retired	45 (15.0)	28 (14)
Hospital ward		
Urology	21 (7.0)	11 (5.5)
Gynaecology	40 (13.3)	21 (10.5)
Gastroenterology	33 (11.0)	25 (12.5)
CCU/ICU/Post CCU	51 (17.0)	27 (13.5)
Surgery/Orthopaedic surgery	89 (29.7)	68 (34)
Neurology	18 (6.0)	10 (5)
Internal	39 (13.0)	32 (16)
Oncology	9 (3.0)	6 (3)
Hospitalization time		
1 week or less	235 (78.3)	148 (74)
More than 1 week	65 (21.7)	52 (26)

Abbreviations: CFA, confirmatory factor analysis; EFA, exploratory factor analysis.

For EFA, the 27‐item scale was delivered to 300 patients who met the inclusion criteria. Skewness, kurtosis, the normal distribution of the data and outliers were assessed. The data were normally distributed. The KMO coefficient was 0.872 and showed the sample size adequacy. Bartlett's test verified that there is not a redundancy between variables that would be summarized with some factors. (df = 78, *p* < 0.001). During the analysis, only 13 of 27 items with the communalities 0.40 and above were entered to the factor analysis. The EFA results showed that there was no cross‐loading between the 13 items. The scree plot showed four factors with eigenvalues equal to one or higher which explained 51.093% of the total variance in the scale. The results of the EFA with Promax rotation indicated that all items were highly loaded on the factors (0.41–0.93), except for one item (item number 2: ‘worried about the absence of family and friends’). The item number 2 was removed due to not loading on the factors. The four factors were labelled according to the items’ content: ‘effective care,’ ‘confidence in the healthcare team,’ ‘emotional enrichment’ and ‘hygienic facilities.’ The results of the EFA are shown in Tables 2 and 3.

**TABLE 2:** The amount of variance explained for the extracted factors in the EFA (*n* = 300).

Items	Initial eigen values			Extraction sums of squared loading before rotated			Extraction sums of squared loading after rotated
	Total	SD%	Cumulative percentage	Total	SD%	Cumulative percentage	Total
1	4.503	34.641	34.61	3.790	29.150	29.150	3.420
2	1.525	11.734	46.375	1.027	7.904	37.054	2.626
3	1.183	9.100	55.475	1.000	7.691	44.744	2.665
4	1.094	8.416	63.892	0.825	6.349	51.093	1.582
5	0.859	6.610	70.502				
6	0.748	5.755	76.257				
7	0.632	4.861	81.118				
8	0.580	4.464	85.583				
9	0.486	3.739	89.322				
10	0.439	3.377	92.698				
11	0.372	2.863	95.561				
12	0.308	2.370	97.931				
13	0.269	2.069	100.000				

Abbreviations: EFA, exploratory factor analysis.

**TABLE 3:** Factors, items factor loading of the SFS in the EFA.

Number of items	Items	Factors			
		1 (effective care)	2 (confidence in the healthcare team)	3 (emotional enrichment)	4 (hygienic facilities)
1	My treatment and care are done on time	0.786			
2	My nurse introduces herself/himself at the beginning of the shift	0.695			
3	Nurses answer my questions	0.687			
4	The hospital staff (doctors, nurses, etc.) pay attention to my requests	0.427			
5	My treatment and care are done without error	0.409			
6	There is close supervision over the work of the hospital staff (doctor, nurse, etc.)		0.961		
7	I am sure that the medical staff (doctors, nurses, etc.) are performing their duties properly		0.550		
8	I'm sure I will recover		0.440		
9	The medical staff (doctors, nurses, etc.) give me hope			0.935	
10	The behaviour of the hospital staff (doctor, nurse, etc.) makes me feel valued			0.690	
11	It is convenient to use the toilets in the ward where I am hospitalized				0.837
12	It is convenient to use the bathroom where I am hospitalized				0.627

Abbreviations: EFA, exploratory factor analysis; SFS, safety feeling scale.

Subsequently, CFA by SEM analysis was conducted to confirm the four‐factor structure of 12‐item scale which was produced by the EFA. Therefore, the 12‐item scale was delivered to 200 new patients. SEM results showed that the model exceeded the sensitivity criteria of the goodness of fit. Eight of nine goodness of fit indices showed an acceptable value (Table 4).

**TABLE 4:** The goodness of fit indices in the CFA (*n* = 200).

Goodness of fit indices	Value	Acceptable threshold
RMSEA	0.081	≤0.1 weak, 0.1–0.08 medium, ≥0.08 good
CMIN/DF	2.97	Acceptable < 5, good < 3
NFI	0.82	≥0.95
PNFI	0.60	<0.5
CFI	0.96	≥0.95
IFI	0.97	≥0.95
RMR	0.059	<0.1
GFI	0.95	≥0.90
AGFI	0.93	≥0.90

Abbreviations: AGFI, Adjusted Goodness of Fit Index; CFA, confirmatory factor analysis; CFI, Comparative of Fit Index; CMIN/DF, Minimum Discrepancy Function by Degrees of Freedom divided; GFI, Goodness of Fit Index; IFI, Tucker‐Lewis Index; NFI, Normed Fit Index; PNFI, Parsimonious Normed Fit Index; RMR, Root Mean Square Residual; RMSEA, Root Mean Square Error of Approximation.

The factor loadings in the model showed that all 12 items of the SFS were highly loaded on the respective factors (0.40–0.84) (Figure S2). Therefore, the construct validity of the SFS scale with 12 items was confirmed in patients who were admitted to the hospital.

#### 3.2.4 Reliability results

Cronbach's alpha coefficient for total scale was 0.74, and for its subscales was between 0.66–078. ICC for the total scale and its subscales were 0.98 and 0.97–0.99 respectively. These results, in addition to the standard error of measurement results, are presented in Table 5.

**TABLE 5:** The reliability results for the SFS and its subscales.

Safety feeling scale (SFS)*	Number of the items	Raw score mean ± SD	Cronbach's alpha	Raw score of the scale	ICC (CI 95%)	SEM	MDC	MDC (%)
SFS	12	38.7 ± 3.7	0.739	12–60	0.98 (0.95–0.98)	0.57	1.57	4.1
Subscale 1	5	15.5 ± 3.3	0.779	5–25	0.99 (0.97–0.99)	0.35	0.96	6.2
Subscale 2	3	10.0 ± 2.4	0.771	3–15	0.98 (0.95–0.98)	0.36	1.01	10.0
Subscale 3	2	6.2 ± 1.15	0.729	2–10	0.97 (0.93–0.98)	0.20	0.55	8.9
Subscale 4	2	6.6 ± 1.8	0.663	2–10	0.98 (0.96–0.99)	0.24	0.66	10.1

Abbreviations: ICC, Intra‐Class Correlation Coefficient; MDC, Minimum Detectable Change; SFS, safety feeling scale; SEM, Standard Error of Measurement.

#### 3.2.5 Feasibility results

The floor and ceiling effects of the scale were 0% and 7% respectively (range 0–0.7). The time required to answer the scale was 8 min (including demographic questions).

#### 3.2.6 Responsiveness results

Based on the results of the MDC, the responsiveness of the scale was excellent (range: 0.55–1.57) (Table 5).

#### 3.2.7 Safety feeling scale (SFS)

After running two phases of the study, the 12‐item SFS with four subscales of ‘effective care’ (5 items), ‘confidence in the healthcare team’ (3 items), ‘emotional enrichment’ (2 items) and ‘hygienic facilities’ (2 items) on a 5‐point Likert scale with 1–5 scores (from always to never) was developed. The total range of raw scale scores is 12–60. After transformation of the scores to the standard score, the scoring of the scale is changed to 0–100. The higher the score, the greater the safety feeling in the patient at the hospital (See File S2).

## 4 DISCUSSION

The aim of the study was to develop and examine psychometric properties of the SFS to measure safety feelings in hospitalized adult patients in our context. At first, the scale was developed by concept analysis, and then the psychometric properties of the scale were examined. The results of the validity and reliability of the scale showed that the SFS is a valid and reliable scale for measuring safety feelings in adult patients in hospitals of our society.

In the phase of the scale development, the items were developed based on a systematic review of qualitative studies, and then we conducted interviews with hospitalized patients. Integrating the results of these two studies brought about clarification of the concept of ‘safety feeling’ in the cultural context of Iran. The result showed the feeling of safety at hospitals is a relative concept with three dimensions and it is perceived by the feeling of protection in a safe place, emotional richness and confidence in the healthcare team and lack of anxiety. It is defined by having needs and expectations met, having positive experiences, receiving human care and family support. A relaxing environment and recourse to spirituality have been created and they lead to a feeling of peace, having a sense of control over the situation, hope for life, adaptability and comfortable spending of hospitalization days.

A review of the literature on similar instruments shows that in most studies, items of safety feeling were extracted only from a review of the literature (Igarashi et al., 2012; Nadighara et al., 2016; Ricci‐Cabello et al., 2016). However, we used a concept analysis method and integrated the results of the systematic review with the qualitative content analysis. Evaluation of similar instruments shows that in one study, authors selected all related qualitative studies but in a different context; inpatient and outpatient settings without considering the target groups (Nadighara et al., 2016). Nevertheless, the concept of safety feeling is dependent on the context of the setting and it can be different in various healthcare settings (Krevers & Milberg, 2014).

In the phase of the psychometrics evaluation, the validity, reliability, feasibility and responsiveness of the scale were supported by different measurements, including examination of face and content validity, item analysis, construct validity, internal consistency and stability reliability, standard error of measurement, floor‐ceiling effect and MDC.

Assessment of the construct validity of the scale by the EFA and Promax rotation showed that there are four factors which explained around 51% of the variance of the scale in a sample of adult patients at hospitals. All items were highly loaded on the factors, except for one item which was removed from the scale based on earlier recommendations in psychometric literature (Kellar & Kelvin, 2013; Keszei et al., 2010; Munro, 2005). It can be discussed that there are different reasons if the item does not get loaded onto the factor, such as inappropriate extraction method or factor rotation, negative coding of the item in the software or when the item has a negative meaning for respondents (34). It seems that in our study there was a negative perception for the removed item by the respondents. The four factors were labelled according to the items’ content: ‘effective care,’ ‘confidence in the healthcare team,’ ‘emotional enrichment’ and ‘hygienic facilities.’ Subsequently, the four‐factor structure of the 12‐item scale was confirmed by CFA. Moreover, factor loadings showed that the results of item‐loadings in factors with two items were satisfactory. Although the number of items was low, they were kept in the factors based on satisfactory loadings and psychometric recommendations (Maryam KHoshbakht et al., 2018).

In the study of Igarashi et al. with cancer patients to examine the factors of security feeling, the five items of the feeling safe were extracted by the EFA and all included in one factor called ‘feeling secure,’ which together explained 73.5% of the variance of feeling safe in cancer patient (22). But, there was no explanation about the type of factor rotation and factor loading values in this study. Until 2013, there was no instrument to measure patient perception of safety‐related issues. Patient measure of safety (PMOS) was one of the first instruments designed in 2013 to measure the feeling of safety in hospitalized patients (41). However, factor analysis was not performed in this study (Giles et al., 2013). Later studies examined the factorial validity of this instrument. One of these studies, after performing the EFA resulted in a large number of the instrument items needing to be revised or removed. In our study, the primary version of the SFS had 84 items, but after the EFA and the rest of our analyses, 72 items were removed.

In our study, reliability of the SFS was examined by internal consistency and stability reliability, and standard error of measurement. All reliability results of the SFS were satisfactory, except for the level of the Cronbach's alpha coefficient of the fourth factor of the scale (Hygienic facilities, *α* = 0.663). It was a little bit less than the acceptance criteria. It can be rationalized that this was due to the low number of the items in the factor, however, in some sources, this is an acceptable value (Maryam KHoshbakht et al., 2018). Furthermore, it is recommended to have more focus on this subscale with different samples in future studies. A review of the literature showed that most studies examined only the internal consistency reliability (Igarashi et al., 2012; Krevers & Milberg, 2014; Nadighara et al., 2016). Only one study, in addition to the internal consistency, examined the stability of the scale and standard error of measurement (Ricci‐Cabello et al., 2016).

Missing data are a challenging issue that occurs due to the failure of some samples to respond to some of the items. It can cause problems in interpretation or understanding. Discomfort with the item asked or non‐usefulness of the item can bring missing data also Donders et al., 2006. For this purpose, the feasibility of the scale was examined, and the results were acceptable.

MDC was calculated to measure the responsiveness of the SFS. In other studies, there was no measurement for the responsiveness of the scale, but in one study, logistic regression was applied to measure the changes in response rate in participants during 2 weeks (Ricci‐Cabello et al., 2016). In the general comparison of the existing measurement with the measurement of the present study, it can be said that most of the studies used a literature review to design items, but in the present study, a combination of literature review and interview was used. Also, we evaluated the validity and reliability of the SFS with a wide range of the psychometric tests, while this was not the case in most of the studies and only a limited number of psychometric indicators of the measurement were examined.

In summary, the SFS is a valid and reliable general self‐report instrument which measures safety feelings of adult patients in hospitals. It is applicable in medical‐surgical wards and ICUs. It can be applied in accreditation hospitals programmes and quality‐of‐healthcare research studies to improve the quality of patient care and allocating necessary resources to meet the patients' needs at the hospital.

### 4.1 Strengthens and limitations of the study

One of the strengths of the present study is the use of a strong methodology, consisting of concept analysis by a hybrid model in three steps (Schwartz‐Barcott, 2000) and running the stages of systematic review and qualitative study continuously to explore the definition and dimensions of the concept of safety feeling in hospitalized patients and releasing the items. Also, we evaluated the validity and reliability of the SFS with a wide range of the psychometric tests. The SFS is a general short scale and applicable for hospitalized adult patients. It can be easily used in different wards of the hospitals, except for psychiatric wards. It is important to mention that this SFS was developed in the context of the healthcare system of Iran. Thus, the applicability of the SFS, is not clear in other cultures. It is suggested to test the applicability of the SFS in other societies in future research.

## 5 CONCLUSIONS

The SFS is a valid and reliable self‐report general instrument with 12 items and four subscales, including ‘effective care,’ ‘confidence in the healthcare team,’ ‘emotional enrichment’ and ‘hygienic facilities.’ It was developed to measure the sense of safety feeling in hospitalized adult patients in our context. It is suggested that it needs to be tested in other societies.

### 5.1 Relevance to clinical practice

The SFS as a short scale can be easily applied in quality‐of‐healthcare research studies and accreditation programmes in hospitals.

## AUTHOR CONTRIBUTIONS

SD: Conceptualization, Methodology, Software, Investigation, Writing—Original Draft, Project administration. MZ: Supervision, Conceptualization. AE: Formal analysis, Validation, Data Curation, Visualization,* He is a statistician on the author team. AA: Supervision, Conceptualization. CR: Writing—Review & Editing, Investigation.

## FUNDING INFORMATION

The authors of this article have not received any funding.

## CONFLICT OF INTEREST STATEMENT

The authors declare no conflict of interest.

## ETHICS STATEMENT

Study was accepted by the research ethics committee of the university (Ethics Code IRSBMU.PHNM1397‐1129).

## Supporting information


File S1.

File S2.

Figures S1 and S2.

## Data Availability

The data that supports the findings of this study are available in the supplementary material of this article. also The data that support the findings of this study are available from the corresponding author upon reasonable request.
