# Supplementary material for: ‘Development and psychometric evaluation of the safety feeling scale in adult patients at hospital: Exploratory sequential mixed method’
Source: Nurs Open. 2023 May 28;10(9):6165–74. doi: 10.1002/nop2.1850 (PMC10416024; doi:10.1002/nop2.1850)
Supplement: Supplementary file 1 — File S1. [file NOP2-10-6165-s003.docx]

Supplementary File 1

**Safety Feeling Scale in Adult Patients at Hospital (English version)**

Dear Respondent

This Scale is developed to measure your safety feeling at the hospital. Please read each of the statements below carefully and choose the best option and insert a check mark (✔) in the box that suits your current situation.

| **Number** | **In response to each of the statements below, please choose only one of the options.** | **Always** | **Often** | **Sometimes** | **Rarely** | **Never** |
| --- | --- | --- | --- | --- | --- | --- |
| 1 | My treatment and care are done on time. |  |  |  |  |  |
| 2 | My nurse introduces herself/himself at the beginning of the shift. |  |  |  |  |  |
| 3 | Nurses answer my questions. |  |  |  |  |  |
| 4 | The hospital staff (doctors, nurses, etc.) pay attention to my requests. |  |  |  |  |  |
| 5 | My treatment and care are done without error. |  |  |  |  |  |
| **Number** | **In response to each of the statements below, please choose only one of the options.** | **Completely Agree** | **Agree** | **Neither Agree Nor Disagree** | **Disagree** | **Completely Disagree** |
| 6 | There is close supervision over the work of the hospital staff (doctor, nurse, etc.). |  |  |  |  |  |
| 7 | I am sure that the medical staff (doctors, nurses, etc.) are performing their duties properly. |  |  |  |  |  |
| 8 | I'm sure I will recover. |  |  |  |  |  |
| 9 | The medical staff (doctors, nurses, etc.) give me hope. |  |  |  |  |  |
| 10 | The behavior of the hospital staff (doctor, nurse, etc.) makes me feel valued. |  |  |  |  |  |
| 11 | It is convenient to use the toilets in the ward where I am hospitalized. |  |  |  |  |  |
| 12 | It is convenient to use the bathroom where I am hospitalized. |  |  |  |  |  |
