# Supplementary material for: ‘Development and psychometric evaluation of the safety feeling scale in adult patients at hospital: Exploratory sequential mixed method’
Source: Nurs Open. 2023 May 28;10(9):6165–74. doi: 10.1002/nop2.1850 (PMC10416024; doi:10.1002/nop2.1850)
Supplement: Supplementary file 2 — File S2. [file NOP2-10-6165-s002.docx]

# Supplementary File 2

# Reporting checklist for quality improvement in health care.

Based on the SQUIRE guidelines.

## Instructions to authors

Complete this checklist by entering the page numbers from your manuscript where readers will find each of the items listed below.

Your article may not currently address all the items on the checklist. Please modify your text to include the missing information. If you are certain that an item does not apply, please write "n/a" and provide a short explanation.

Upload your completed checklist as an extra file when you submit to a journal.

In your methods section, say that you used the SQUIREreporting guidelines, and cite them as:

Ogrinc G, Davies L, Goodman D, Batalden P, Davidoff F, Stevens D. SQUIRE 2.0 (Standards for QUality Improvement Reporting Excellence): revised publication guidelines from a detailed consensus process

|  |  | Reporting Item | Page Number |
| --- | --- | --- | --- |
| **Title** |  |  |  |
|  | [#1](https://www.goodreports.org/reporting-checklists/squire/info/#1) | Development and Psychometric Evaluation of the Safety Feeling Scale in Adult Patients at Hospital | 1 |
| **Abstract** |  |  |  |
|  | [#02a](https://www.goodreports.org/reporting-checklists/squire/info/#02a) | This study aimed to develop and examine psychometric properties of the Safety Feeling Scale (SFS) in adult patients to assess their sense of safety during a hospital stay. | 1 |
|  | [#02b](https://www.goodreports.org/reporting-checklists/squire/info/#02b) | **Background:** Patients’ perceptions of feeling safe at the hospital impacts their recovery, well-being and satisfaction.  **Design**: mixed-method design. A SQUIRE checklist was used.  **Methods:** This is a study with two phases of scale development and evaluation of psychometric properties of the scale. In the first phase, the concept of “safety feeling” was analyzed using a hybrid model. Thus, a systematic review and then a qualitative study with hospitalized patients (n=31) were conducted by conventional content analysis. In the psychometric phase, factorial validity, reliability, feasibility and responsiveness of the scale were evaluated by different tests in various samples.  **Patient or Public Contribution:** All participants were informed about the aim of the study, voluntary participation (Interview) and the confidentiality of the information. They were also informed that they could withdraw at any time of the study and written informed consent forms were obtained from all of them.  **Results:** After integrating the results of the systematic review and qualitative study, a scale item pool with 84 items was developed. In the psychometric phase, 12 items with four factors were specified; “effective care,” “confidence in the healthcare team,” “emotional enrichment,” and “hygienic facilities,” explaining 51% of the total variance of the scale. They were confirmed by confirmatory factor analysis. Internal consistency and stability of the scale were satisfactory. Feasibility and responsiveness were also acceptable.  **Conclusions:** SFS is a valid and reliable self-report scale with 12-item for measuring the sense of safety feeling among hospitalized patients in our context  **Relevance to clinical practice**: This scale can be applied in accreditation hospital programs and quality-of-healthcare research studies. | 1&2 |
| **Introduction** |  |  |  |
| Problem description | [#3](https://www.goodreports.org/reporting-checklists/squire/info/#3) | Patients are at the core of care in clinical settings. Delivering safe healthcare services to the patients is a highly important issue and fundamental principle in healthcare settings (Williams, 2008). According to the World Health Organization, “patient safety” means prevention and reduction of risks, errors and harms that can occur during the health care of the patient. There is no specific theory in nursing about patient safety. But, there are several theories in other disciplines, including "Bowlby's an Ethical Approach" in 1973 (Bowlby, 1973), Fry's "Safety year?, the Therapeutic Milieu" in 1987, and Rachman's "Safety Signals” in 1994 (Woody & Rachman, 1994) (Fry, 1987). Measurement of patient safety is a key element in securing and delivering high-quality healthcare services. Also, this is a multi-step, systematic, and multidisciplinary goal in healthcare settings (Dabaghi et al., 2020; Dehghan Nayeri et al., 2016; Shali et al., 2016). Providing patient safety, facilitates the provision of safe care for both patients and healthcare personnel (Mhammadi, 2017). | 2 |
| Available knowledge | [#4](https://www.goodreports.org/reporting-checklists/squire/info/#4) | A review of the literature shows that there are various studies on patient safety in hospital settings to prevent or decrease accidents and medical errors. Evidence shows that “culture” in different communities can influence patients’ perceptions and thus patients’ safety, so there is a priority for research in this area (Dabaghi et al., 2020; Ricci-Cabello et al., 2016). Unfortunately, adverse events and negative consequences for patients in medical settings still occur frequently It seems that the complexity of the healthcare environment and multiple environmental factors, impact the provision of safe care (Lee et al., 2010). There are studies on patient safety culture among healthcare providers and influencing factors, such as the relationship between nurse and patient, hand hygiene, and falling out of bed (Quillivan et al., 2016; Wang & Tao, 2017; Zwijnenberg et al., 2016). However, patients’ perceptions play an important role in their awareness of the overt and covert problems in the healthcare settings (Lasiter, 2011; Mollon, 2014). “Being safe” is not the same as “feeling safe” (Lasiter, 2011). Therefore, studies were conducted to understand the patients’ experiences and perspectives on the concept of safety (Lasiter, 2011; Mollon, 2014; Russell, 1999). Initially, it was assumed that “safety” gains its meaning from the Intensive Care Units (ICUs), which have sophisticated monitoring equipment and patients have severe injuries (Hupcey, 2000; Russell, 1999). Over time, more studies were conducted in various hospital wards, such as surgery and obstetric wards (Lee et al., 2010; Nilsson, 2014; Vaismoradi et al., 2011). In these studies, the authors tried to define the concept of safety using different qualitative methods. But, there are many disparities in the definitions at interdisciplinary and intradisciplinary level. For instance, many studies have used the terms “safety” and “security” interchangeably, but there is a border between the meanings of these two terms. Clarification of patient safety is an essential prerequisite to produce a valid and reliable culturally-appropriate instrument, and then to develop health interventions and to promote patients’ safety at clinical settings. However, there are a few instruments for the measurement of the sense of patients’ safety in hospitals. | 3 |
| Rationale | [#5](https://www.goodreports.org/reporting-checklists/squire/info/#5) | We found only five instruments in this area; the Scale of Security Feeling in medical-surgical wards (Nadighara et al., 2016), Patient Measure of Safety (POMS) (Giles et al., 2013a), Sense of Security in Care - Patients’ Evaluation (SEC-P) (Igarashi et al., 2012) and the Feelings of Support and Security in cancer care department (Krevers & Milberg, 2014). Appraisal of selected instruments by COSMIN checklist (COnsensus-based Standards for the selection of health status Measurement Instruments) showed that these instruments are used in specific wards, such as oncology or internal medicine and surgery, and also in different societies. None of them was valid for our context and no study examined all aspects of feeling safe in detail. Moreover, we identified tan overlap between the wo words of safety and security in these studies. Furthermore, most of them used only a review of the literature for development of an instrument. Finally, we concluded that in none of the studies, the psychometric phases of the instrument were fully performed. | 4 |
| Specific aims | [#6](https://www.goodreports.org/reporting-checklists/squire/info/#6) | The aim of this study was to develop a valid and reliable general scale to measure safety feelings in hospitalized adult patients in the cultural context of Iran. | 4 |
| **Methods** |  |  |  |
| Context | [#7](https://www.goodreports.org/reporting-checklists/squire/info/#7) | This is a mixed-method study with exploratory sequential design. It was performed in two different phases: scale development and psychometric properties evaluation | 4 |
| Intervention(s) | [#08a](https://www.goodreports.org/reporting-checklists/squire/info/#08a) | In the first phase of the study, a concept analysis was done on the concept of “safety feeling” using a hybrid model by Shwartz-Barcott and Kim in three steps, consisting of theoretical stage, fieldwork and final analysis. Also, We wanted to answer two questions base on the literature: “what is the explicit meaning of safety feeling?” and “what is the boundary between “safety feeling” and “security feeling” at hospital?.” In this step, a systematic review was conducted on the qualitative studies which focused on the patients’ perceptions and/or experiences about the features of safety feeling at the hospital. Results of the systematic review and qualitative study was integrated, and then a scale item pool was produced. In the second phase, the validity and reliability of the scale were examined by different tests and samples. | 5&6 |
| Intervention(s) | [#08b](https://www.goodreports.org/reporting-checklists/squire/info/#08b) | Hospitalized patients | 11 |
| Study of the Intervention(s) | [#09a](https://www.goodreports.org/reporting-checklists/squire/info/#09a) | n/a ( our study was scale development and we didn’t have any intervention) |  |
| Study of the Intervention(s) | [#09b](https://www.goodreports.org/reporting-checklists/squire/info/#09b) | n/a ( our study was scale development and we didn’t have any intervention) |  |
| Measures | [#10a](https://www.goodreports.org/reporting-checklists/squire/info/#10a) | In the psychometric phase, factorial validity, reliability, feasibility and responsiveness of the scale were evaluated by different tests in various samples. | 11&12&13 |
| Measures | [#10b](https://www.goodreports.org/reporting-checklists/squire/info/#10b) | n/a ( our study was scale development and we didn’t have any intervention) |  |
| Measures | [#10c](https://www.goodreports.org/reporting-checklists/squire/info/#10c) | We used several approaches in stages (Polit & Yang in psychometric phase and Schwartz-Barcott, Graneheim & Lundman in phase qualitative phase) | 6&7 |
| Analysis | [#11a](https://www.goodreports.org/reporting-checklists/squire/info/#11a) | Meta-Analyses, concept analysis and scale development | 6&7 |
| Analysis | [#11b](https://www.goodreports.org/reporting-checklists/squire/info/#11b) | Methods for understanding variation within the data, including the effects of time as a variable |  |
| Ethical considerations | [#12](https://www.goodreports.org/reporting-checklists/squire/info/#12) | This study was accepted by the research ethics committee of the university (Ethics Code XX). All participants were informed about the aim of the study, voluntary participation and the confidentiality of the information. They were also informed that they could withdraw at any time of the study and written informed consent forms were obtained from all of them. | 4 |
| **Results** |  |  |  |
|  | [#13a](https://www.goodreports.org/reporting-checklists/squire/info/#13a) | n/a ( our study was scale development and we didn’t have any intervention) |  |
|  | [#13b](https://www.goodreports.org/reporting-checklists/squire/info/#13b) | All the details of the results (separately for each stage) have been described (from page number 12 to 9) | 12-14 |
|  | [#13c](https://www.goodreports.org/reporting-checklists/squire/info/#13c) | n/a ( our study was scale development and we didn’t have any intervention) |  |
|  | [#13d](https://www.goodreports.org/reporting-checklists/squire/info/#13d) | n/a ( our study was scale development and we didn’t have any intervention) |  |
|  | [#13e](https://www.goodreports.org/reporting-checklists/squire/info/#13e) | n/a ( our study was scale development and we didn’t have any intervention) |  |
|  | [#13f](https://www.goodreports.org/reporting-checklists/squire/info/#13f) | The data were treated and outliers were removed and missing values were replaced by the series mean in the SPSS version 20. In both EFA and CFA, the amount of missing values were acceptable (less than 5%). | 9 |
| **Discussion** |  |  |  |
| Summary | [#14a](https://www.goodreports.org/reporting-checklists/squire/info/#14a) | The aim of the study was to develop and examine psychometric properties of the SFS to measure safety feelings in hospitalized adult patients in our context. At first, the scale was developed by concept analysis, and then the psychometric properties of the scale were examined. The results of the validity and reliability of the scale showed that the SFS is a valid and reliable scale for measuring safety feelings in adult patients in hospitals of our society. | 14 |
| Summary | [#14b](https://www.goodreports.org/reporting-checklists/squire/info/#14b) | One of the strengths of the present study, is the use of a strong methodology, consisting of concept analysis by a hybrid model in three steps (Schwartz-Barcott, 2000) and running the stages of systematic review and qualitative study continuously to explore the definition and dimensions of the concept of safety feeling in hospitalized patients and releasing the items. Also, we evaluated validity and reliability of the SFS with a wide range of the psychometric tests. The SFS, is a general short scale and applicable for hospitalized adult patients. It can be easily used in different wards of the hospitals, except for psychiatric wards. | 17 |
| Interpretation | [#15a](https://www.goodreports.org/reporting-checklists/squire/info/#15a) | n/a ( our study was scale development and we didn’t have any intervention) |  |
| Interpretation | [#15b](https://www.goodreports.org/reporting-checklists/squire/info/#15b) | the SFS is a valid and reliable general self-report instrument which measures safety feelings of adult patients in hospitals. It is applicable in medical-surgical wards and Intensive Care Units. It can be applied in accreditation hospitals programs and quality-of-healthcare research studies to improve the quality of patient care and allocating necessary resources to meet the patients’ needs at the hospital. | 17 |
| Interpretation | [#15c](https://www.goodreports.org/reporting-checklists/squire/info/#15c) | The SFS as a short scale can be easily applied in quality-of-healthcare research studies and accreditation programs in hospitals. | 18 |
| Interpretation | [#15d](https://www.goodreports.org/reporting-checklists/squire/info/#15d) | . It is important to mention that this SFS was developed in the context of the health care system of Iran. Thus, the applicability of the SFS, is not clear in other cultures. It is suggested to test the applicability of the SFS in other societies in future research. | 17 |
| Interpretation | [#15e](https://www.goodreports.org/reporting-checklists/squire/info/#15e) | n/a ( our study was scale development and we didn’t have any intervention) |  |
| Limitations | [#16a](https://www.goodreports.org/reporting-checklists/squire/info/#16a) | . It is important to mention that this SFS was developed in the context of the health care system of Iran. Thus, the applicability of the SFS, is not clear in other cultures. | 17 |
| Limitations | [#16b](https://www.goodreports.org/reporting-checklists/squire/info/#16b) | n/a (In this study, an attempt has been made to use scientific and up-to-date approaches. In addition, the research team had experience in patient safety and scale development( |  |
| Limitations | [#16c](https://www.goodreports.org/reporting-checklists/squire/info/#16c) | n/a (we had no limitation) |  |
| Conclusion | [#17a](https://www.goodreports.org/reporting-checklists/squire/info/#17a) | The Safety Feeling Scale (SFS) is a valid and reliable self-report general instrument with 12 items and four subscales, including “effective care,” “confidence in the health care team,” “emotional enrichment,” and “hygienic facilities.” It was developed to measure the sense of safety feeling in hospitalized adult patients in our context. It is suggested that it needs to be tested in other societies. | 18 |
| Conclusion | [#17b](https://www.goodreports.org/reporting-checklists/squire/info/#17b) | n/a (scale reliability was evaluate) |  |
| Conclusion | [#17c](https://www.goodreports.org/reporting-checklists/squire/info/#17c) | The SFS as a short scale can be easily applied in quality-of-healthcare research studies and accreditation programs in hospitals. | 18 |
| Conclusion | [#17d](https://www.goodreports.org/reporting-checklists/squire/info/#17d) | It was developed to measure the sense of safety feeling in hospitalized adult patients in our context. It is suggested that it needs to be tested in other societies. | 18 |
| Conclusion | [#17e](https://www.goodreports.org/reporting-checklists/squire/info/#17e) | It is suggested that it needs to be tested in other societies. | 18 |
| **Other information** |  |  |  |
| Funding | [#18](https://www.goodreports.org/reporting-checklists/squire/info/#18) | n/a |  |

None The SQUIRE 2.0 checklist is distributed under the terms of the Creative Commons Attribution License CC BY-NC 4.0. This checklist can be completed online using <https://www.goodreports.org/>, a tool made by the [EQUATOR Network](https://www.equator-network.org) in collaboration with [Penelope.ai](https://www.penelope.ai)
