# Supplementary material for: ‘Development and psychometric evaluation of the safety feeling scale in adult patients at hospital: Exploratory sequential mixed method’
Source: Nurs Open. 2023 May 28;10(9):6165–74. doi: 10.1002/nop2.1850 (PMC10416024; doi:10.1002/nop2.1850)
Supplement: Supplementary file 3 — Figures S1 and S2. [file NOP2-10-6165-s001.docx]

**Phase 1:**

Scale Development

**Concept analysis:**

**a.** Theoretical stage (literature review; n=25 articles)

**b.** Field work stage

(Conventional content analysis; n=31participants)

**c.** Final analysis stage

(Data integration)

**Scale item-pool:**

**Feeling Safety Scale**

(n=94 items)

F

**Reliability:**

**a.** Internal consistency

(n=30 patients)

**b.** ICC (n=30 patients)

**C.** SEM (n=30 patients)

**Item analysis**

(n=30 patients)

**Safety Feeling Scale (SFS):**

12-item, Four subscales

**Responsiveness:**

Minimum Detectable Change (MDC)

**Feasibility:**

**a.** Floor-ceiling effect

**b.** Time to response

**Phase 2:**

Psychometric

Evaluation

**Figure 1**. The phases of the study for scale development and psychometric analysis

**Content validity:**

**a.** CVR (n=15 experts)

**b.** CVI (n=10 experts) and calculation of Kappa coefficient

**Construct validity:**

**a.** EFA (n=300 patients)

**b.** CFA (n=200 patients)

**Face validity:**

**a.** Quantitative with Item Impact Scale (n=10 patients);

**b.** Qualitative

(n=10 patients)

**
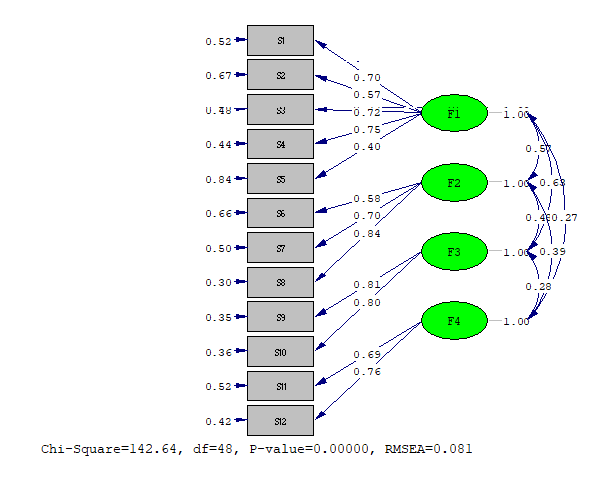
**

**Figure 2:** The diagram of the Safety Feeling Scale by the Structural Equation Modeling (n=200)
